# Sustainable and green extraction of citrus peel essential oil using intermittent solvent-free microwave technology

**DOI:** 10.1186/s40643-025-00885-6

**Published:** 2025-05-29

**Authors:** Yuhao Xie, Longdi Zhang, Wenting Wu, Jiahao Xie, Boliang Gao, Yiwen Xiao, Du Zhu

**Affiliations:** 1 Jiangxi Province Key Laboratory of Natural Microbial Medicine Research, Key Laboratory of Microbial Resources and Metabolism of Nanchang City, College of Life Sciences, Jiangxi Science and Technology Normal University, Nanchang, Jiangxi 330013 People’s Republic of China; 2 Jiangxi Center of Medical Device Testing, Nanchang, Jiangxi 330029 People’s Republic of China; 3 Jiangxi Province Key Laboratory of Biodiversity Conservation and Bioresource Utilization, School of Life Sciences, Jiangxi Normal University, Nanchang, Jiangxi 330022 People’s Republic of China

**Keywords:** Solvent-free microwave extraction, Essential oil, Nanfeng tangerine, Orange peel, Energy consumption

## Abstract

In the present study, a novel technology called intermittent solvent-free microwave extraction (ISFME), which integrates intermittent microwave treatment with solvent-free extraction, was proposed. Taking Nanfeng citrus peel as the research object, the extraction efficiency and composition of its essential oil were evaluated and compared with conventional hydrodistillation (HD) and solvent-free microwave extraction (SFME). Results from single-factor experiments and response surface method optimization indicate that the optimal parameters for SFME are a power of 660 W, a duration of 20 min, and a water content of 60%, yielding an essential oil output of 3.47%. In contrast, the optimal parameters for ISFME, determined through single-factor experiments, are a moisture content of 50%, a power setting of 380 W, a reaction time of 6 min, a 15 min interval, and 4 reaction cycles, resulting in an essential oil yield of 3.51%. Notably, the extraction efficiencies of ISFME and SFME are significantly superior to that of HD, which requires 240 min and yields only 3.28% essential oil. Furthermore, ISFME demonstrates comparable performance to SFME in terms of reaction time, extraction rate, and oil quality, while operating at a substantially lower power requirement of 380 W compared to the 660 W needed for SFME. Additionally, the carbon dioxide emissions and energy consumption associated with ISFME are markedly lower than those of traditional methods, amounting to only one-third of the energy consumption of SFME. These findings underscore that ISFME not only enhances the efficiency and quality of essential oil extraction but also minimizes environmental impact, thereby highlighting its significant advantages in terms of sustainability and ecological responsibility.

**Graphical Abstract:**

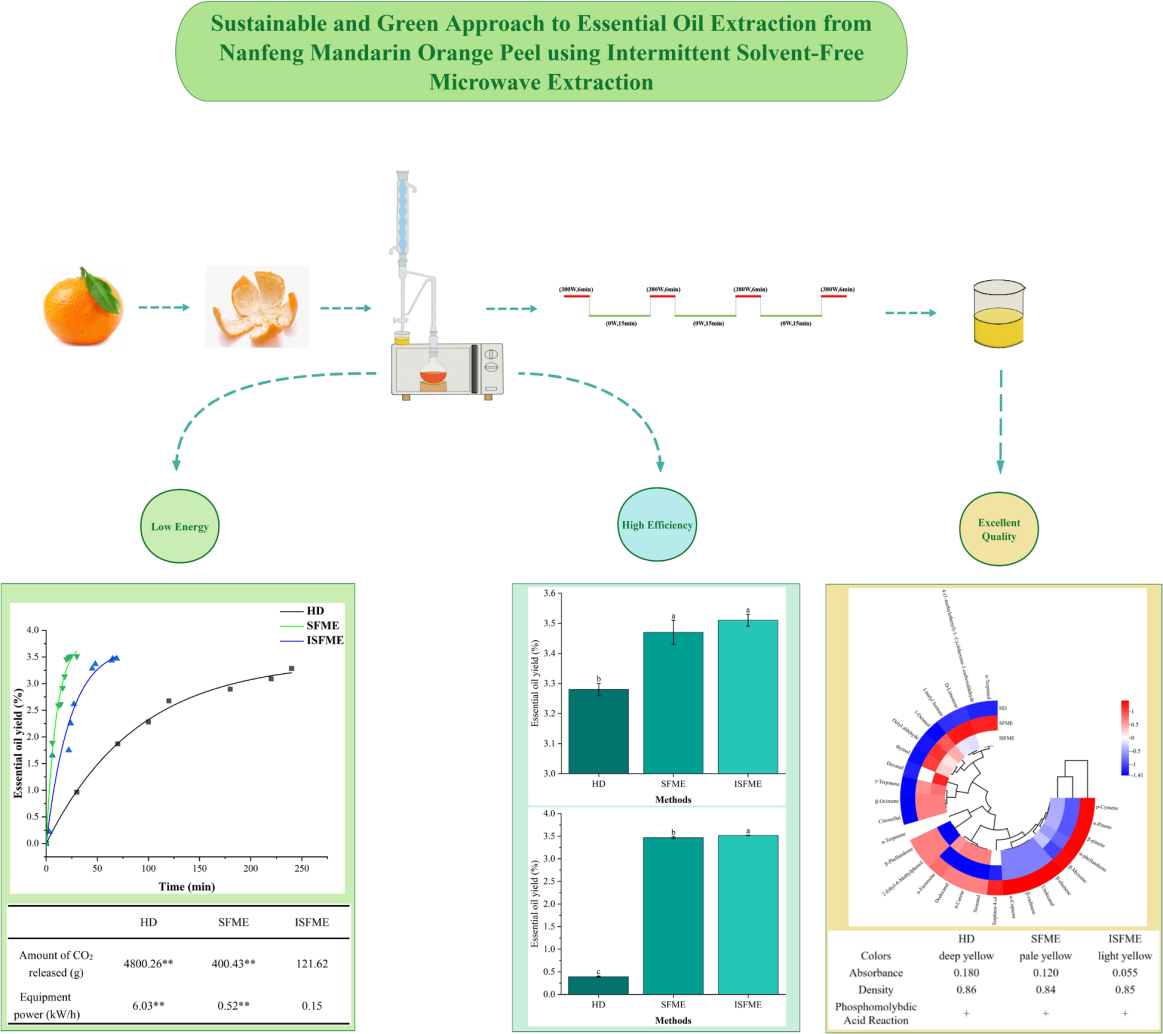

**Supplementary Information:**

The online version contains supplementary material available at 10.1186/s40643-025-00885-6.

## Introduction

Essential oils, commonly known as volatile oils, are intricate mixtures primarily consisting of monoterpenes (such as limonene, pinene, and myrcene), sesquiterpenes, and their oxidized derivatives. These components are predominantly found in the stems, roots, leaves, flowers, fruits, and other parts of aromatic plants (Joseph et al. 2023). In recent decades, essential oils have gained considerable significance across various fields, serving as antioxidants and preservatives that provide alternatives to synthetic chemicals (Bhandari et al. 2021; Falleh et al. 2020). They are extensively utilized in industries including food, pharmaceuticals, and cosmetics.

Essential oils can be extracted through various methods, including traditional hydrodistillation (HD), solvent extraction, and supercritical fluid extraction (Jing et al. 2019). However, these above-mentioned techniques are associated with several drawbacks, such as prolonged extraction times, high energy consumption, and low yields of essential oil. While microwave-assisted hydrodistillation demonstrates remarkable efficiency in extracting essential oils (Mollaei et al. 2019), the significant addition of water to this extraction system results in the hydrolysis of certain components. Consequently, there is an urgent need to explore alternatives to traditional essential oil extraction methods. Lucchesi et al. (2004) introduced Solvent-free Microwave Extraction (SFME), which integrates traditional microwave-assisted extraction (MAE) with dry distillation. Notably, SFME eliminates the need for any solvents, including water, by utilizing the moisture inherent in the extracted material itself. Compared to conventional extraction methods, SFME significantly reduces energy consumption by avoiding solvent heating and mitigates potential issues related to odor and skin irritation that may arise from the hydrolysis of certain components. Furthermore, while traditional HD requires approximately 4 h for extraction, SFME achieves this in about 30 min. Consequently, SFME has gained popularity as a more economical, direct, and rapid green method for preparing essential oils (Liu et al. 2018; Pu et al. 2023). For instance, Liu et al. (2018) employed SFME to extract essential oils from *Cinnamomum camphora* leaves, which not only yielded a higher quantity of essential oils compared to HD but also produced a greater concentration of oxygenated components. Similarly, Pu et al. (2023) combined SFME with enzyme pretreatment to extract essential oils from the fresh rhizomes of *Acorus tatarinowii* Schott, demonstrating that SFME enhanced extraction efficiency and increased the active ingredient β-asarone in the essential oils by 18.06% relative to HD.

Citrus, a member of the Rutaceae family (Pei et al. 2023), is the most productive fruit plant globally. It is abundant in nutrients such as carbohydrates, minerals, vitamins, and dietary fiber (Lu et al. 2023). The consumption and processing of citrus fruits generate substantial amounts of orange peel. Despite their high potential for utilization, orange peels are often discarded, leading to resource waste and environmental pollution. Existing research indicates that orange peels are rich in essential oils, which exhibit significant biological activities, including the inhibition of bacteria and fungi, as well as robust antioxidant properties encompassing free radical scavenging, iron-reducing antioxidant activity, and the inhibition of lipid peroxidation (Cebi and Erarslan 2023; Li et al. 2022). Furthermore, essential oils extracted from orange peels has been shown to significantly inhibit the activities of collagenase and tyrosinase (Prommaban and Chaiyana 2022). Consequently, orange peel essential oil is widely utilized in spices, food preservatives, and cosmetics (Talib Al-Sudani et al. 2024). Notably, it has demonstrated considerable potential in inhibiting viral attachment and replication, thereby mitigating severe symptoms associated with COVID-19 infection (Arena et al. 2021). Therefore, research focusing on the extraction, development, and utilization of orange peel essential oils is crucial for maximizing the use of orange peel resources and minimizing environmental pollution. To date, several studies have reported on the extraction of citrus essential oils using SFME (Teigiserova et al. 2021; Tsai et al. 2017; Zhou et al. 2023; Mahato et al. 2019). Taktak et al. (2021) conducted an extraction of essential oils from *Citrus sinensis* peel, comparing five extraction methods: HD, salt-assisted hydrodistillation, ultrasound-assisted extraction, enzyme-assisted extraction, and SFME. Their findings revealed that SFME yielded the highest amount of essential oil while consuming the least energy and requiring the shortest extraction time. Additionally, Wei et al. (2023) employed SFME to extract essential oil from finger citron peel, demonstrating that this oil possesses excellent antioxidant, acetylcholinesterase inhibitory, and antibacterial properties.

Although SFME offers numerous advantages, its continuous high-power microwave heating can lead to the overheating of plant raw materials, potentially resulting in gelatinization or coking. These effects can adversely impact the extraction efficiency and quality of essential oils. Meanwhile, continuous microwave treatment may lead to excessive energy loss. In contrast, the discontinuous (or segmented continuous) characteristics of intermittent microwave allows for a more uniform distribution of moisture and temperature within the material during the intermittent processing phases, thereby preventing overheating. To date, intermittent microwaves have been effectively employed for drying food and agricultural products, yielding positive results (Kumar et al. 2016; Silveira et al. 2024). However, this method has not yet been applied to the field of essential oil extraction.

To address the above-mentioned challenges in SFME, herein, we propose the integration of intermittent microwave treatment with solvent-free extraction, resulting in an improved method termed intermittent SFME (ISFME). To further evaluate the efficacy of ISFME, this study utilized the peel of Nanfeng mandarin, a unique variety indigenous to the Nanfeng region, which boasts a planting history exceeding 1300 years and historically has been recognized as a royal tribute. We employed traditional HD extraction, alongside SFME and our proposed ISFME, to investigate the extraction efficiencies of essential oil from orange peels. Our comprehensive analysis included comparisons of extraction rates, physical and chemical properties, component composition, energy consumption, and CO_2_ emissions associated with the extraction of orange peel essential oil. Furthermore, we constructed an extraction kinetic model. The results indicate that ISFME outperforms the other methods in terms of both essential oil extraction rate and quality, while also demonstrating significantly lower power consumption and CO_2_ emissions. These findings suggest that the improved ISFME method holds substantial promise for future applications in essential oil extraction.

## Experimental

### Experimental materials

Nanfeng mandarin was sourced from Nanfeng County in Jiangxi Province, China (27°12′ N, 116°23′ E) on November 23rd, 2022. Fresh orange peels were peeled and stored in the refrigerator at – 18 °C prior to use. Four strains (*Staphylococcus aureus*, *Salmonella typhimurium*, *Shigella flexneri* and *Bacillus cereus*) were purchased from Shanghai Luwei Technology Co., Ltd (Shanghai, China).

### Apparatus and procedure

The SFME equipment is primarily adapted from a household microwave oven (G90 F25 CN3L-C2, Galanz Company, Foshan, China), which operates at a fixed frequency of 2450 MHz (Figure S1). In this setup, a round-bottom flask is positioned within the microwave oven and connected to a condenser via a directional tube, which subsequently leads to a Clevenger Type Apparatus (Liu et al. 2018). During the extraction process, water vapor carrying essential oils is condensed into a distillate through the condenser and collected using an oil–water separator. The microwave oven has a rated output power of 700 W, with the actual power consumption during extraction being dynamically adjustable based on specific requirements through power feedback and control.

### Essential oil extraction

#### Essential oil extraction process

Drawing upon the methodology of Liu et al. (2018) with slight modifications tailored to the specifics of this experiment. About 200 g of visually selected based on quality criteria Nanfeng mandarin peels were selected and processed in a grinder (BF-02, BenChen, Hebei, China) for 2 min (1500 r/min). Subsequently, the crushed fresh peel weighed equal to 20 g of dry weight was transferred into a 500 mL round-bottom flask and the flask was placed in a microwave oven to facilitate the extraction process. Various power levels and reaction times were employed during the microwave treatment. Upon completion of the extraction, the essential oils were collected and dehydrated with waterless Na_2_SO_4_ and then stored in a refrigerator at 4 °C prior to analysis. The yield of essential oil was calculated according to the following formula:

$$\text{Yield of essential oil}(\text{\%})=\frac{\text{Quality of essential oil }(\text{g})\times 100\text{\%}}{\text{Quality of dry sample }(\text{g})}$$

#### The processes of SFME and ISFME for essential oil extraction

The SFME method employed a preliminary single-factor experimental design to analyze the influence of varying moisture contents (40, 50, 60, 70, and 80%), microwave irradiation times (5, 10, 15, 20, and 25 min), and microwave irradiation powers (120, 220, 380, 540, 700, and 860 W) on the yield of citrus essential oils. This analysis established the ranges for each factor to be utilized in the subsequent response surface methodology. Further optimization to determine the optimal conditions for SFME of citrus essential oils was conducted using Design-Expert software (Stat-Ease, Inc., Ver. 8.0, Minneapolis, MN, USA).

Based on the SFME operating conditions obtained above, the optimal ISFME conditions to maximize the yield of citrus essential oils were determined by investigating various factors, including moisture content (40, 50, 60, 70, and 80%), microwave single-interval duration (5, 10, 15, 20, 25, and 30 min), microwave single-heating time (3, 4, 5, 6, 7, 8, and 9 min), microwave irradiation power (120, 220, 380, 540, and 700 W), and the number of microwave heating cycles (1, 2, 3, 4, and 5 times).

### Comparison of environmental impacts of different extraction methods for Nanfeng tangerine essential oil

Refer to Romdhane et al. (Benmoussa et al. 2018), with appropriate modifications. The yield of Nanfeng mandarin peel essential oil, the extraction time, and the amount of CO_2_ released (assuming an emission of approximately 800 g of CO_2_ per kWh of electricity consumed) were calculated.

Reduction in electricity consumption (kWh) = electricity consumption before improvement (kWh)—electricity consumption after improvement (kWh)

$${\text{CO}}_{2}\text{ emissions}=\frac{\text{Time of essential oil extraction }(\text{h}) \times \text{ Equipment power }(\text{Kw}/\text{h})}{800} (\text{g})$$

### Extraction kinetics

This study analyzed the extraction kinetics models associated with the HD, SFME, and ISFME methods, drawing upon Fick's first law of diffusion (Chen et al. 2021). The extraction time and essential oil yield were plotted as the horizontal and vertical axes, respectively, to construct the extraction kinetic curves, which can be mathematically represented by the following equation:

According to mass transfer theory, the rate of change of solute concentration in the extract phase can be expressed as:

$$\frac{dY}{dt}=ka\left({Y}_{e}-Y\right)$$

Rewrite the equation as:

$$\frac{dY}{{Y}_{e}-Y}=kadt$$

Integrate both sides, with the integration intervals being from 0 to t (for time) and from 0 to *Y* (for concentration):

$${\int }_{0}^{Y}\frac{1}{{Y}_{e}-{Y}^{\prime}}d{Y}^{\prime}={\int }_{0}^{t}kad{t}^{\prime}$$

Calculate the integrals, yielding:

$$-\text{ln}\left(\frac{{Y}_{e}-Y}{{Y}_{e}}\right)=kat$$

Further rearrange to obtain:

$$\frac{{Y}_{e}-Y}{{Y}_{e}}=\text{exp}\left(-kat\right)$$

$$Y={Y}_{e}\left[1-\text{exp}\left(-kat\right)\right]$$

In practical applications, the thickness *L* of the extract phase may affect the mass transfer rate. To account for this factor, we can multiply the mass transfer coefficient *k* by *L* to obtain the overall mass transfer rate constant *kLa*.

Therefore, the final extraction kinetics formula is:

$$Y={Y}_{e}\left[1-\text{exp}\left(-kLa\cdot t\right)\right]$$

*Y* and *Y*_e_ represent the essential oil yield at any given time and at equilibrium, respectively, both measured in mg/g. *k* represents the mass transfer coefficient, and t is the extraction time, expressed in min. *L* represents Thickness or characteristic length of the extracted phase. *a* represents contact area between extractive phase and feedstock phase.

### Gas chromatography-mass spectrometry (GC–MS) analysis

Chemical analysis of Nanfeng mandarin essential oil was conducted using a Thermo Trace-1300 ISQ Mass Chromatograph (Thermo Fisher Scientific Technologies, Waltham, MA, USA) coupled with a TG-5MS chromatography column (30 m × 0.25 mm × 0.25 μm). The GC–MS parameters were set as follows: a 1 μL injection volume, a split ratio of 20:1, an injector temperature of 220 °C, and a flow rate of 1.0 mL/min, with high-purity helium as the carrier gas. The temperature program initiated at 50 °C, subsequently increased to 160 °C at a rate of 8 °C/min, and was maintained for 8 min. This was followed by a further increase to 260 °C at the same rate, which was held for an additional 12 min. For the mass spectrometry conditions, an electron impact (EI) ionization source was employed, set at an ionization voltage of 70 eV, with both the ion source and transfer line temperatures maintained at 280 °C. Standard tuning was performed using FULLSCAN mass scanning, accompanied by a solvent delay of 2.5 min and a scan range of 20 to 600 atomic mass units (amu).

In the component analysis, volatile compounds were identified using the NIST Mass Spectral Library (NIST 17) and the NIST Mass Spectral Search Program (version 2.2), following the methodology outlined by Li et al. (2022). Matches were accepted when the similarity index exceeded 85%. Additionally, a series of homologous n-alkanes (C8-C40) were employed to determine relative retention indices (RIs). The relative concentrations of individual components were calculated through peak area normalization.

### Measurement of basic physicochemical properties of Nanfeng Tangerine essential oil

The density of Nanfeng mandarin essential oil was determined using the gravimetric-volumetric method. For the phosphomolybdic acid reaction, a 10% phosphomolybdic acid–ethanol solution was prepared and sprayed onto the sample for testing. Upon heating to a high temperature, the development of a blue-gray color indicates a positive result (+), while the absence of color change signifies a negative result (−). This method is specifically employed for the detection of terpenes, esters, and alcohols (Vasta and Sherma 2008).

### Evaluation of bacteriostatic activity and determination of minimum inhibitory concentration (MIC)

The antimicrobial effect of essential oil extracted by ISFME method against six pathogenic bacteria was tested by Oxford cup method (Wang et al. 2022). 2% agar Luria–Bertani (LB) medium was used as plate substrate and 1.5% agar LB medium was accessed for incubation of pathogenic bacteria for 24 h and then poured into the plates. The plates were allowed to stand for 20 min. Four Oxford cups were placed evenly in each plate and each cup was punched with 200 µl of samples (two control volatile oil samples, positive control chloramphenicol 400 µg/ml and negative control pure water respectively). Three parallel groups were made for each pathogen and the media plates were incubated in a biochemical incubator at 37 °C for 24 h. At the end of the incubation, the size of the diameter of the circle of inhibition was observed and measured, and the results were averaged over replicates to assess the inhibitory effect of the volatile oils. The MIC determination of the antimicrobial experiments was carried out in multiple groups at essential oil concentrations. The result was taken as the MIC at the essential oil concentration corresponding to the smallest inhibitory circle that appeared (Bai et al. 2023).

### Data analysis

Statistical analyses were conducted using SPSS software (version 22.0; SPSS Inc., Chicago, IL, USA), employing single-factor analysis of variance (ANOVA) followed by Tukey's post-hoc test for multiple group comparisons. Differences were deemed statistically significant at a *P*-value of less than 0.05 (denoted as *: *P* < 0.05). Graphs were generated using Origin 2021 software (OriginLab, Northampton, MA, USA). Results are presented as mean ± standard deviation (SD).

## Results and discussion

### Effect of microwave irradiation power, time and moisture on the yield of essential oils by SFME method

**Fig. 1:**
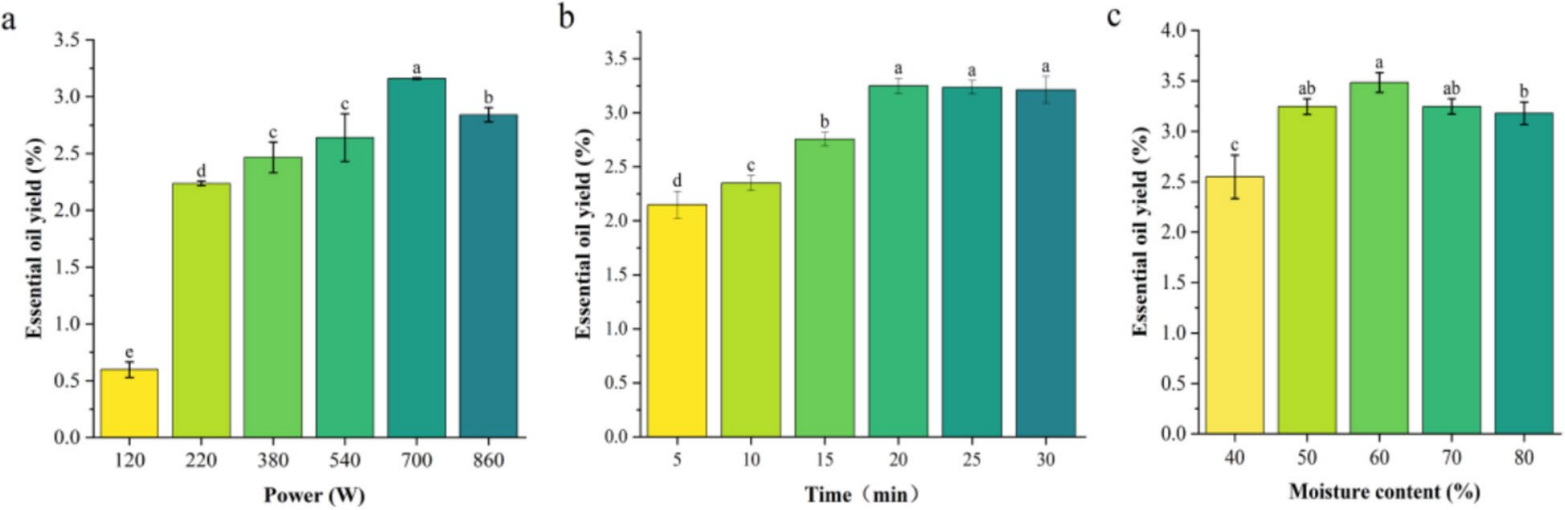
The three main factors affecting the yield of essential oil in the SFME method. **a**: Power (120, 220, 380, 540, 700, 860 W), **b**: Time (5, 10, 15, 20, 25, 30 min) and **c**: Moisture content (40, 50, 60, 70, 80%)

Microwave irradiation power is one of the key variables affecting the yield of essential oils, which can influence the amount of heat generated inside the material and the speed of rotation of polar molecules in the material. As the microwave irradiation power increases, so does the essential oil extraction rate (Desai and Parikh 2015). As illustrated in Fig. 1a, the essential oil yield peaked at 700 W (3.16%) and then began to decline, which was significantly different from all other powers. This decline can be attributed to high-power microwave irradiation, which causes localized charring and carbonization of the plant material, thereby, inhibiting the release of volatile components (Peng et al. 2021; Singh Chouhan et al. 2019). The duration of microwave irradiation is a critical variable influencing the yield of essential oils. Some studies have demonstrated that prolonged application of microwave radiation enhances the absorption of microwave energy, thereby facilitating the more efficient separation of essential oils from raw materials (Hosseini et al. 2016). However, it has also been established that excessive exposure to microwaves may adversely affect both the quality and yield of essential oils (Kusuma et al. 2018). Prolonged exposure to radiation can lead to excessive energy accumulation, resulting in tissue degradation, charring, and the loss of certain volatile components (Chen et al. 2010). Consequently, a response surface optimization was conducted within a microwave power range of 540 to 860 W. After determining the optimal microwave power, identified as 700 W, an investigation was initiated to assess the impact of varying microwave radiation times on essential oil yield. As illustrated in Fig. 1b, the essential oil yield exhibited a marked increase within the 5 to 20 min range, while remaining largely unchanged beyond this period. The optimal range for microwave radiation time in subsequent experiments was established at 15 to 25 min. SFME combines microwave heating with distillation at atmospheric pressure. The internal heating of the water in the orange peel promotes the swelling of the peel, rupturing glandular cavities, and the release of oil-containing tissues (Kratchanova et al. 2004). It has been demonstrated that a higher water content in orange peel, correlates with increased essential oil extraction (Ma et al. 2012). Figure 1c illustrates the variation in essential oil yield as a function of water content, which ranges from 40 to 80%. Notably, when the water content increased from 40 to 60%, the essential oil yield exhibited a significant rise, peaking at 3.48%. However, as the water content continued to increase beyond this point, the essential oil yield gradually declined. Consequently, the range of water content selected for the optimization experiments was established between 50 and 70%.

### Optimization of SFME parameters by response surface methodology (RSM)

**Fig. 2:**
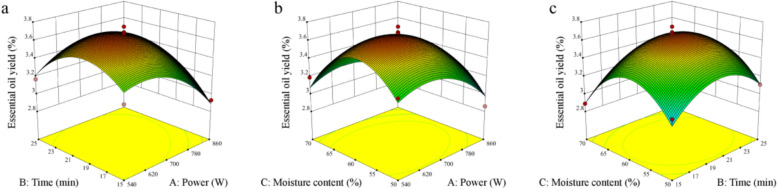
Response surface plots of the effect of SFME method variables on the yield of Nanfeng mandarin orange. **a**: Interaction between Power and Time on the extraction yields of Nanfeng mandarin orange, **b**: Interaction between Power and Moisture content on the extraction yields of Nanfeng mandarin orange, **c**:Interaction between Time and Moisture content on the extraction yields of Nanfeng mandarin orange

A RSM involving three factors and three levels was conducted using Design Expert 10.0 software (Fig. 2) to further investigate the interactions among three parameters: microwave power (X_1_), microwave reaction time (X_2_), and moisture content (X_3_) on the yield of essential oil extracted from Nanfeng mandarin oranges. The results obtained from the quadratic polynomial equation modeling these interactions are summarized in Table 1. Additionally, the analysis of variance (ANOVA) for the model is presented in Table 2.

**Table 1:** Results of response surface experimental design for SFME methodology

No	Power (W)	Time (min)	Moisture content (%)	Essential oil yield (%)
1	540 (− 1)	15 (− 1)	60 (0)	3.26
2	860 (+ 1)	15 (− 1)	60 (0)	2.93
3	540 (− 1)	25 (+ 1)	60 (0)	3.17
4	860 (+ 1)	25 (+ 1)	60 (0)	3.34
5	540 (− 1)	20 (0)	50 (− 1)	3.32
6	860 (+ 1)	20 (0)	50 (− 1)	2.86
7	540 (− 1)	20 (0)	70 (+ 1)	3.19
8	860 (+ 1)	20 (0)	70 (+ 1)	2.85
9	700 (0)	15 (− 1)	50 (− 1)	3.11
10	700 (0)	25 (+ 1)	50 (− 1)	3.11
11	700 (0)	15 (− 1)	70 (+ 1)	2.89
12	700 (0)	25 (+ 1)	70 (+ 1)	2.91
13	700 (0)	20 (0)	60 (0)	3.69
14	700 (0)	20 (0)	60 (0)	3.75
15	700 (0)	20 (0)	60 (0)	3.68
16	700 (0)	20 (0)	60 (0)	3.55
17	700 (0)	20 (0)	60 (0)	3.67

**Table 2:** ANOVA of regression model of SFME method

Source	Sum of squares	df	Mean Square	*F*-Value	*p*-value Prob > F	
Model	1.52	9	0.17	12.67	0.0015	**
A-Power	0.12	1	0.12	8.62	0.0218	*
B-Time	0.014	1	0.014	1.08	0.3329	
C-moisture content	0.039	1	0.039	2.93	0.1305	
AB	0.062	1	0.062	4.68	0.0673	
AC	0.0036	1	0.0036	0.27	0.6197	
BC	0.0001	1	0.0001	0.0075	0.9335	
A^2^	0.21	1	0.21	15.46	0.0057	
B^2^	0.31	1	0.31	23.23	0.0019	**
C^2^	0.65	1	0.65	48.3	0.0002	**
Residual	0.094	7	0.013			
Lack of Fit	0.072	3	0.024	4.53	0.0894	not significant
Pure Error	0.021	4	0.0053			
Cor Total	1.62	16				

“*” indicates a significant effect on the results (*p* < 0.05), “**” indicates a highly significant effect on the results (*p* < 0.01)

Utilizing Design Expert 10.0 software, a multivariate regression analysis was conducted on the data presented in Table 2, resulting in the following quadratic regression equation:

$${\text{Y}} = {3}.{67} - 0.{\text{12X}}_{{1}} + 0.0{\text{43X}}_{{2}} - 0.0{\text{7X}}_{{3}} + 0.{\text{12X}}_{{1}} {\text{X}}_{{2}} + 0.0{\text{3X}}_{{1}} {\text{X}}_{{3}} + 0.00{\text{5X}}_{{2}} {\text{X}}_{{3}} - 0.{\text{22X}}_{{1}}^{{2}} - 0.{\text{27X}}_{{2}}^{{2}} - 0.{\text{39X}}_{{3}}^{{2}}$$

The *p*-values were utilized to assess the significance of the coefficients, thereby clarifying the interactions among the model variables. Specifically, *p* < 0.05 indicates a highly significant difference, while *p* < 0.0001 denotes an extremely significant difference, and *p* > 0.01 suggests no significant difference. As presented in Table 2, the regression model demonstrates high significance with a *p*-value of 0.0015 (*p* < 0.01). In contrast, the lack of fit term is not significant (*p* = 0.0894, *p* > 0.05), indicating a good fit for the model. The coefficient of determination (R^2^) of the equation is 0.9422, whereas the adjusted coefficient of determination (R^2^_adj_) is 0.8678, signifying that the model accounts for 86.78% of the variation in the response variable. The P-value for the first-order term A (microwave power) in the model equation is less than 0.05, demonstrating that microwave power significantly influences the yield of essential oil. Furthermore, the order of influence of each first-order factor on essential oil yield is as follows: microwave power > moisture content > reaction time. Both second-order terms B^2^ and C^2^ have *p*-values of less than 0.05 and less than 0.01, respectively, confirming their significance. This underscores the complexity of essential oil yield variation, as the effects of microwave power, reaction time, and moisture content on yield from orange peels are not merely linear but involve significant response surface interactions. The data indicates that the regression equation exhibits a good fit and reliability, making it suitable for theoretical predictions regarding essential oil extraction from Nanfeng mandarin. The optimization of essential oil yield using Design Expert 10.0 software identified the optimal conditions for achieving maximum oil yield: a microwave power of 634.70 W, a reaction time of 17.99 min, and a moisture content of 59.86%. Under these conditions, the model predicts a theoretical maximum essential oil yield of 3.65%.

To validate the accuracy and practicality of the model, while also considering experimental convenience, we adjusted the parameters of the essential oil extraction process to a microwave power of 660 W, a microwave reaction time of 20 min, and a moisture content of 60%. Under these modified conditions, the essential oil yield obtained was 3.47%, which closely aligns with the theoretically predicted value. This finding indicates that the model is suitable for optimizing the SFME method.

### Effect of five main factors content on the yield of essential oils by ISFME method

The ISFME method involves transforming a single variable, specifically the time of microwave radiation, into three distinct variables: microwave single reaction time, interval time, and the number of reactions. This approach differs from the SFME method by preventing sample overheating while maintaining equilibrium between heat and mass transfer processes. Additionally, the ISFME method has been shown to enhance the yield of bioactive compounds (Chumnanpaisont et al. 2014).

**Fig. 3:**
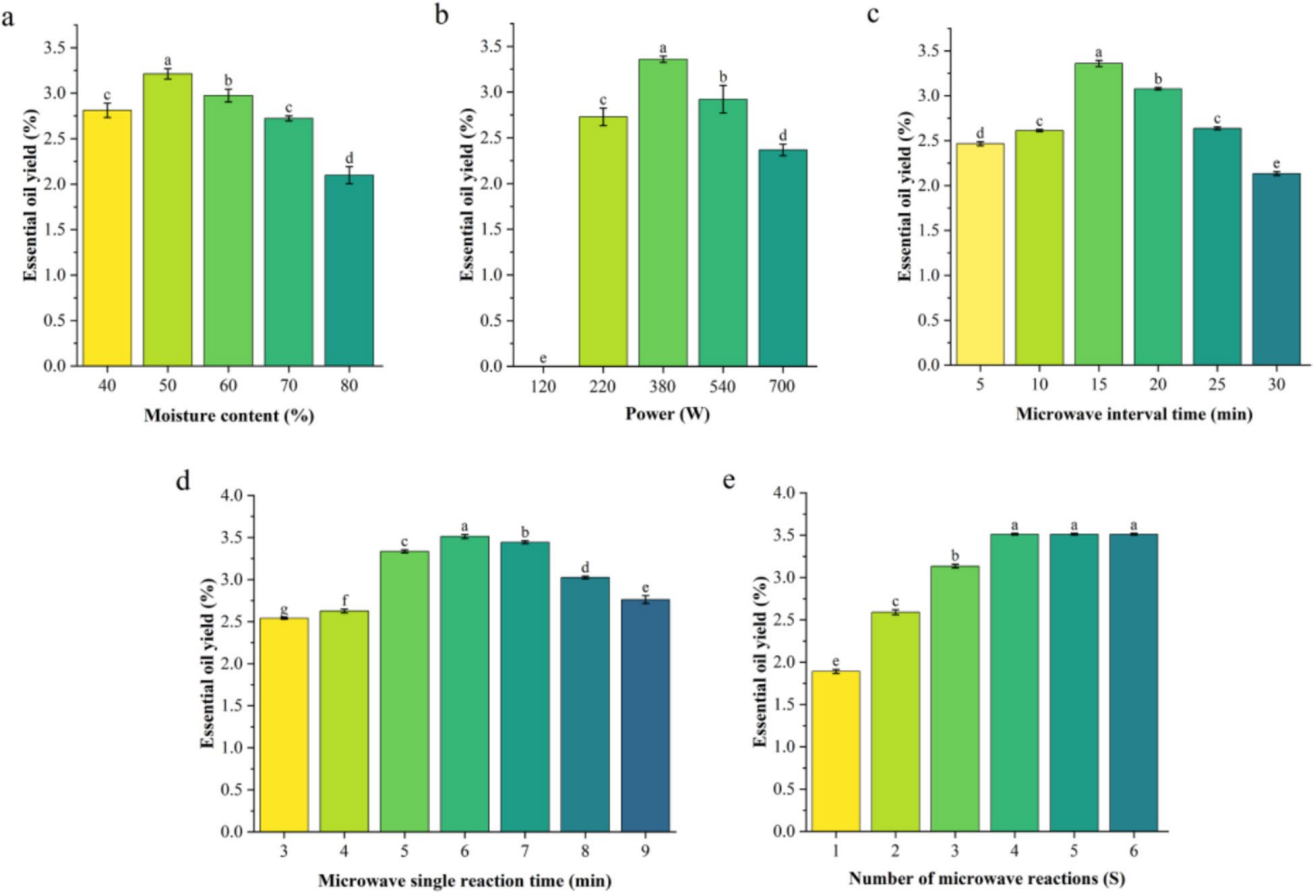
The five main factors affecting the yield of essential oil in the ISFME method. **a**: moisture content (40, 50, 60, 70, 80%), **b**: Power (120, 220, 380, 540, 700 W), **c**: Microwave interval time (5, 10, 15, 20, 25, 30 min), **d**: Microwave single reaction time (3, 4, 5, 6, 7, 8, 9 min) and **e**: Number of microwave reactions (1, 2, 3, 4, 5, 6 S)

In the case of SFME, water content is a significant influencing factor in ISFME. This study focused on essential oil extraction at a power setting of 660 W, employing a single microwave reaction time of 5 min, an interval of 15 min, and a total of four reactions. As illustrated in Fig. 3a, yield increased significantly with increasing water content and then decreased significantly, reaching a maximum at 50% (3.21%). It was also noted that the rate of essential oil extraction gradually decreased as moisture content continued to rise, a trend consistent with observations made in the SFME method. Subsequently, an investigation was conducted to ascertain the impact of microwave power on the yield of essential oils. As illustrated in Fig. 3b, the relationship between essential oil yield and power showed a tendency of increasing and then decreasing, and reached a peak at 380 W. The results showed that the microwave power had a significant effect on the essential oil yield. Through the significance analysis, it can be inferred that the microwave power has a significant effect on the essential oil yield. At a microwave power of 120 W, the production of essential oil was negligible, likely due to the insufficient capacity of low microwave power to effectively disrupt the essential oil storage tissue. As the power was increased to 380 W, the essential oil yield reached its maximum value, indicating that microwave energy in this power range was effective in extracting essential oils. However, when the power continued to increase to 540 W and above, the essential oil yield decreased significantly. Conversely, at a microwave power of 380 W, the essential oil yield reached its maximum value, which was significantly lower than that required for SFME at 660 W, as optimized by response surface methodology. This finding suggests that the ISFME method can achieve an optimal essential oil extraction rate through the use of intermittent multiple microwave radiation. Additionally, reducing the power required for microwave radiation can effectively prevent tissue charring, degradation, and loss of volatiles, thereby enhancing the quality of the essential oil. Following this, the effect of microwave single interval time was investigated, with results presented in Fig. 3c. The yield of essential oil showed a significant increase correlated with the prolongation of the microwave single interval time. Notably, applying a 15 min microwave interval time resulted in relatively high yields of essential oils. However, increasing the interval time from 15 to 30 min resulted in a notable decline in essential oil yield. An optimal microwave single interval time can effectively prevent the deterioration of quality or efficiency of the target due to overheating, as noted by (Vorhauer et al. 2019). When the microwave single reaction time was varied, as illustrated in Fig. 3d, the yield of essential oils increased significantly as the microwave reaction time increased and then decreased. A relatively high essential oil yield of 3.51% was achieved at a microwave single reaction time of 6 min, surpassing the optimal essential yield of 3.47% obtained through SFME. This indicates that the ISFME method is an effective technique for extracting essential oil from orange peel. The yield of essential oil demonstrated a gradual increase with the number of microwave reactions, as depicted in Fig. 3e. Reaching a peak yield of 3.51% after four microwave reactions. Beyond this point, increasing the number of microwave reactions did not result in significant changes in essential oil yield, suggesting that the maximum yield had been attained, with water vapor evaporation occurring after four reactions. Although this process required a considerable amount of energy, it was determined that conducting four microwave reactions represented the optimal experimental outcome from an energy-saving perspective. The highest yield of essential oil was achieved under optimal conditions, which were established through a one-way optimization of water content, microwave power, single interval and single reaction time.

### Optimization of ISFME parameters by response surface methodology (RSM)

**Fig. 4:**
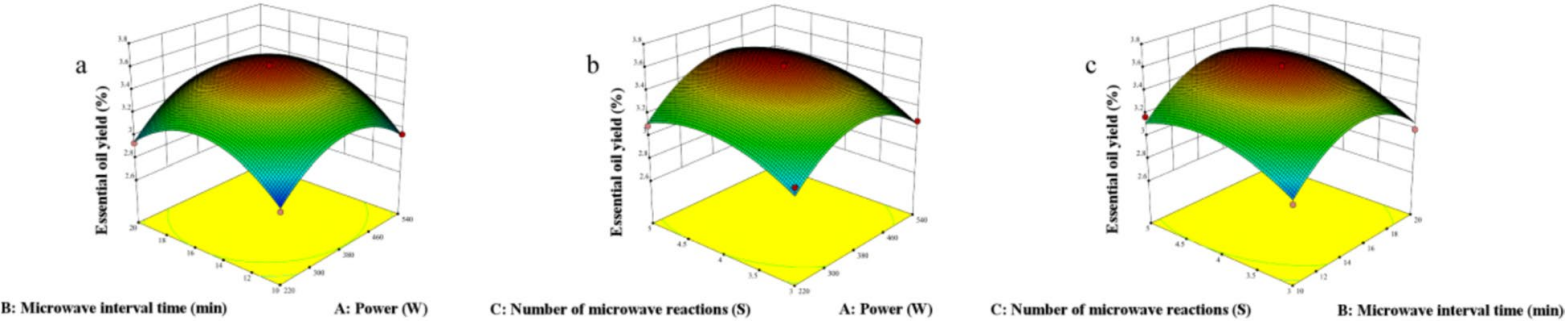
Response surface plots of the effect of ISFME method variables on the yield of Nanfeng mandarin orange. **a**: Interaction between Power and Microwave interval time on the extraction yields of Nanfeng mandarin orange, **b**: Interaction between Power and Number of microwave reactions on the extraction yields of Nanfeng mandarin orange, **c**:Interaction between Microwave interval time and Number of microwave reactions on the extraction yields of Nanfeng mandarin orange

Plackett–Burman experiments were performed on the five factors of ISFME (Figure S2). From the figure, it can be found that the adjustment of Power, Microwave interval time and Number of microwave reactions can significantly affect the essential oil yield, which is the key to optimize the extraction efficiency. Therefore, RSM analyses of Power (X_1_), Microwave interval time (X_2_) and Number of microwave reactions (X_3_) were also carried out using Design Expert 10.0 software (Fig. 4). The results for different interactions are summarised in Table 3. In addition, the ANOVA of the model is shown in Table 4.

**Table 3:** Results of response surface experimental design for ISFME methodology

No	Power (W)	Microwave interval time (min)	Number of microwave reactions (S)	Essential oil yield (%)
1	220(− 1)	10(−1)	4(0)	2.82
2	220(− 1)	20(+ 1)	4(0)	2.93
3	220(− 1)	15(0)	3(− 1)	3.02
4	220(− 1)	15(0)	5(+ 1)	3.09
5	380(0)	10(−1)	3(− 1)	2.88
6	380(0)	20(+ 1)	3(− 1)	2.99
7	380(0)	10(−1)	5(+ 1)	3.17
8	380(0)	20(+ 1)	5(+ 1)	3.33
9	380(0)	15(0)	4(0)	3.58
10	380(0)	15(0)	4(0)	3.59
11	380(0)	15(0)	4(0)	3.50
12	380(0)	15(0)	4(0)	3.58
13	380(0)	15(0)	4(0)	3.58
14	540(+ 1)	10(− 1)	4(0)	2.94
15	540(+ 1)	20(+ 1)	4(0)	3.18
16	540(+ 1)	15(0)	3(− 1)	3.07
17	540(+ 1)	15(0)	5(+ 1)	3.24

**Table 4:** ANOVA of regression model of ISFME method

Source	Sum of squares	df	Mean square	F-value	p-value Prob > F	
Model	1.17	9	0.13	31.01	< 0.0001	Significant
A-Power	0.042	1	0.042	10.02	0.0158	*
B-Microwave interval time	0.049	1	0.049	11.67	0.0112	*
C-Number of microwave reactions	0.095	1	0.095	22.74	0.002	**
AB	4.03E−03	1	4.03E−03	0.96	0.3588	
AC	3.10E−03	1	3.10E−03	0.74	0.4175	
BC	8.35E−04	1	8.35E−04	0.2	0.6684	
A^2^	0.37	1	0.37	87.34	< 0.0001	
B^2^	0.4	1	0.4	94.57	< 0.0001	
C^2^	0.12	1	0.12	28.17	0.0011	
Residual	0.029	7	4.18E−03			
Lack of fit	0.023	3	7.74E−03	5.11	0.0745	Not significant
Pure Error	6.06E−03	4	1.51E−03			
Cor Total	1.2	16				

“*” indicates a significant effect on the results (*p* < 0.05), “**” indicates a highly significant effect on the results (*p* < 0.01)

Utilizing Design Expert 10.0 software, a multivariate regression analysis was conducted on the data presented in Table 4, resulting in the following quadratic regression equation:

$${\text{Y}} = {3}.{57}\,{ + }\,0.0{\text{72X}}_{{1}} + 0.0{\text{78X}}_{{2}} + 0.{\text{11X}}_{{3}} + 0.0{\text{32X}}_{{1}} {\text{X}}_{{2}} + 0.0{\text{28X}}_{{1}} {\text{X}}_{{3}} + 0.0{\text{14X}}_{{2}} {\text{X}}_{{3}} - 0.{\text{29X}}_{{1}}^{{2}} - 0.{\text{31X}}_{{2}}^{{2}} - 0.{\text{17X}}_{{3}}^{{2}}$$

As shown in Table 4, the regression model was highly significant at *p* < 0.0001. In contrast, the lack of fit term was not significant (*p* = 0.0745, *p* > 0.05), indicating that the model was well fitted. The coefficient of determination (R^2^) of the equation was 0.9755, while the adjusted coefficient of determination (R^2^_adj_) was 0.9441, indicating that the model explained 94.41% of the variance in the response variable. The first-order term C (Number of microwave reactions) in the model equation had the smallest *P*-value of 0.002 (*p* < 0.01), indicating that this factor had the most significant effect on essential oil yield. In addition, the order of influence of each first-order factor on essential oil yield was: Number of microwave reactions > Microwave interval time > Power. This illustrates the complexity of the variation in essential oil yield because the effects of Power, Microwave interval time and Number of microwave reactions on essential oil yield are not only linear but also involve response surface interactions. The data indicate that the regression equation has good fit and reliability and is suitable for theoretical prediction of essential oil extraction from Nanfeng mandarin. The essential oil yield was optimised using Design Expert 10.0 software and the best conditions for obtaining the highest essential oil yield were determined: Power of 391.43 W, Microwave interval time of 15.593 min and Number of microwave reactions of 4.239. Under these conditions, the model predicted the theoretical maximum essential oil yield. Under these conditions, the model predicted a theoretical maximum essential oil yield of 3.598%. In order to verify the accuracy and practicality of the model, and taking into account the convenience of the experiment, we adjusted the parameters of the essential oil extraction process by ISFME method with Power of 380 W, Microwave interval time of 15 min, and Number of microwave reactions of 4 times. The modified conditions matched the conditions of the one-way experiment and the essential oil yield (3.51) was very close to the theoretical prediction. This result indicates that the model is suitable for optimising the SFME method.

Overall, the ISFME method yielded higher essential oil outputs compared to that SFME method. This improvement was achieved by modifying several variables and conducting the extraction at low microwave power. The optimal conditions identified included 50% water content, a microwave power of 380 W, a single microwave interval of 15 min, a single microwave reaction time of 6 min, and four microwave reactions, resulting in an optimal essential oil yield of 3.51%. Bustamante et al (2016). used the Microwave-assisted hydro-distillation method to extract orange peel essential oil, with the optimal conditions being: a first step where the biomass consisting of a 1:1.5 (waste orange peel:water) mixture is irradiated using 785 W for 5 min, followed by a second step in which the system is irradiated using 250 W for 15 min (maintaining a constant pressure of 300 mbar throughout the entire irradiation process). The essential oil yield was 1.8%, significantly lower than the yield obtained by the ISFME method in our experiment (3.51%), and the required power was significantly higher than that of the ISFME method (380 W), indicating that the ISFME method has great potential for application in the field of essential oil extraction.

### Comparing essential oil yields of HD, SFME and ISFME methods

**Fig. 5:**
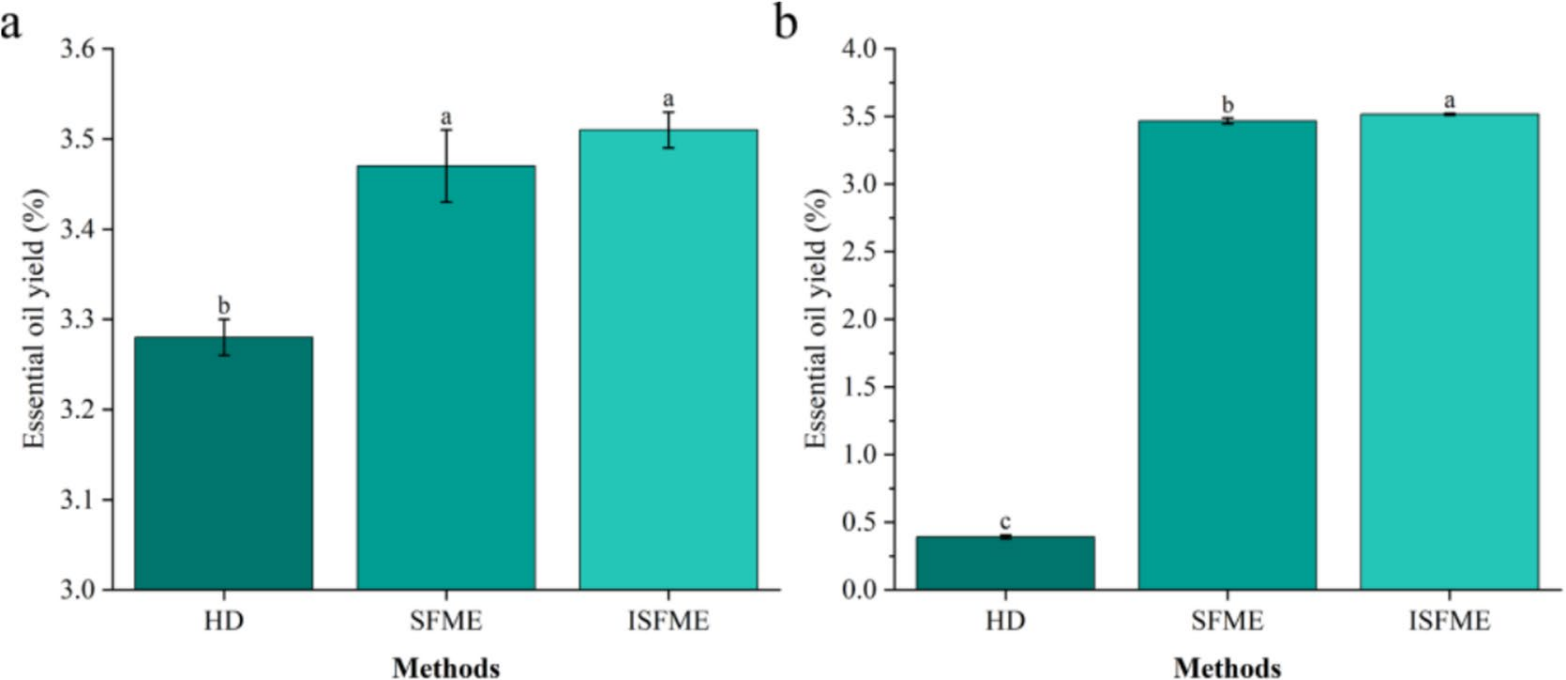
Comparison of the yields of essential oils from various extraction methods (HD, SFME and ISFME) under optimal conditions (**a**) and the same extraction time (**b**)

**Fig. 6:**
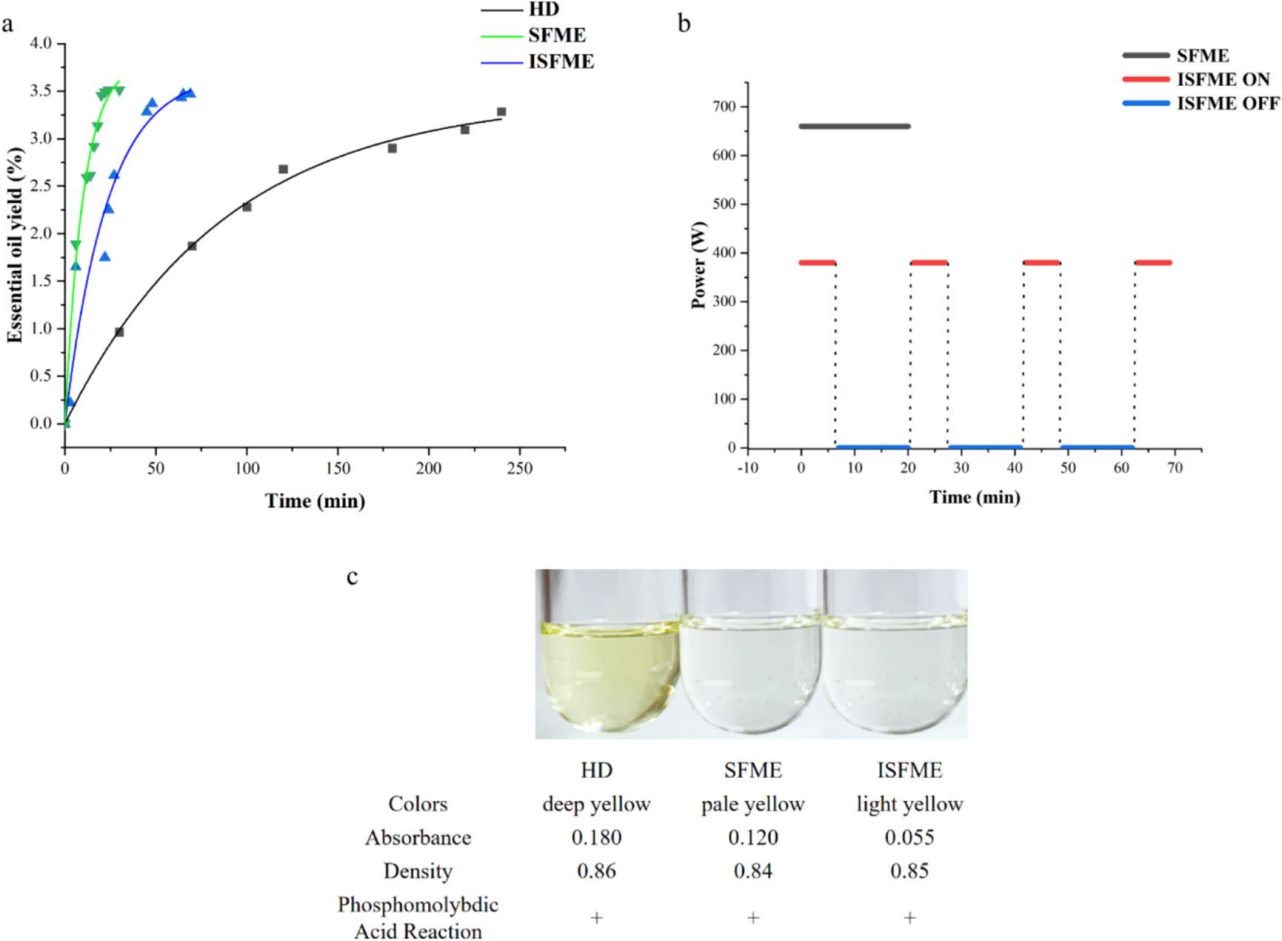
**a**: Extraction kinetics curves of different extraction methods. **b**: Processes SFME and ISFME (ON: Microwave on state, OFF: Microwave off state). **c**: Physical and chemical properties of different extraction methods

Currently, the primary extraction methods for essential oils include HD. This study compares the essential oil extraction outcomes of three methods under optimal conditions, as depicted in Fig. 5a. The maximum essential oil yields achieved by the HD, SFME, and ISFME methods were 3.28%, 3.47%, and 3.51%, respectively. Notably, the ISFME method yielded the highest concentration of essential oil, demonstrating a significant difference from the traditional methods. The kinetic models clearly illustrate the disparities in extraction rates and yields among the different methods, as shown in Fig. 6a. The HD method achieved the maximum essential oil yield at 240 min, which is 10 times that of ISFME (with an actual effective extraction time of 24 min for ISFME) and 12 times that of SFME.This underscores the superiority of both SFME approaches over the traditional HD methods in terms of essential oil yield and extraction time. Similar findings have been reported in previous studies investigating the extraction of essential oils from various citrus fruits. In a comparative study, Taktak et al. (2021) examined the efficacy of the SFME method for extracting essential oils from citrus peels, contrasting it with the HD. The results revealed that the SFME showed highest yield (3.58 ± 0.05) and consuming shorter time (30 min), Additionally, an analysis of the essential oil extraction results from the three methods at a consistent extraction time of 24 min (Fig. 5b) indicates that the extraction rate of the HD method was only 0.4%, significantly lower than those of the ISFME and SFME methods, thereby supporting the aforementioned conclusions.

Table 5 presents the experimental values and fitted mass transfer kinetic parameters for the equilibrium points achieved during the extraction of essential oil from Nanfeng mandarin peels using the HD, SFME, and ISFME methods. The R^2^ values for all three extraction methods exceed 0.95, indicating a strong correlation between the experimental data and the mass transfer kinetic models. This finding suggests that the selected mass transfer kinetic models can accurately characterize the extraction kinetics of essential oil from Nanfeng mandarin peels. As evident from Table 5, the *k*_L_ · *a* values of the SFME extraction process are notably higher compared to those of the HD method and marginally superior to those of the ISFME method. This disparity is attributed to the enhancement of the diffusion coefficient of the essential oils with increasing temperature. Specifically, during the SFME extraction, the continuous microwave radiant heating of orange peels rapidly elevates the internal cellular temperature, which in turn boosts the diffusion coefficient of the essential oils. Consequently, the oils diffuse more swiftly out of the cells and volatilize alongside the water vapor, leading to a more efficient extraction of the essential oils with the water vapor (Handayani et al. 2008). Therefore, the mass transfer rate of essential oil extracted by SFME method was faster. Although the time required to reach the equilibrium point was longer for the ISFME method than for the SFME method, it was found by analyzing the optimal extraction conditions of the two methods that the ISFME method showed better performance in terms of microwave power and essential oil yield. As shown in Figs. 6a and 6b, its essential oil yield obtained with only 380 W was significantly higher than the result of 660 W for the SFME method. From this perspective, the ISFME method represents a more efficient process for extracting essential oil from Nanfeng mandarin peels.

**Table 5:** Mass transfer kinetic parameters of Nanfeng tangerine essential oil extraction

Method	The mass-transfer kinetic parameters					
	*Y*_e_ experiment (%)	*Y*_e_ fitted (%)	Standard error	*k*_L_ *a*	Standard error	R^2^
HD	3.28	3.26	0.021	0.021	0.003	0.9587
SFME	3.47	3.44	0.031	0.197	0.027	0.9746
ISFME	3.51	3.49	0.018	0.124	0.020	0.9931

In comparing the characteristics of essential oil extracted from Nanfeng mandarin peel using various methods, we observed significant variations in color and absorbance. As depicted in Fig. 6c, the oil extracted via the HD method exhibited the darkest color, followed by that extracted using the SFME method, while the oils obtained from the ISFME methods appeared lighter. The absorbance measurements taken at 405 nm using a UV–Vis spectrophotometer revealed values of 0.180, 0.120 and 0.055 for the essential oils extracted by the HD, SFME, and ISFME methods, respectively. Additionally, substantial differences were noted in density measurements, with the oils ranked in descending order of density as follows: HD > ISFME > SFME. This finding underscores that the choice of extraction method not only influences the color and absorbance of the essential oil but also significantly affects its physical density. There was a significant difference in the colour of the essential oils extracted by the HD method and the SFME and ISFME methods, as shown by the dark yellow colour of the essential oils of the HD method and the pale yellow colour of the SFME and ISFME methods. Considering that there was no significant difference in the density of the essential oils between the three methods mentioned above, it can be excluded that the difference in the percentage of substances in the essential oils extracted by the three methods resulted in the difference. It is assumed that the main reason for the difference in colour may be the longer reaction time and high temperature of the HD method compared with the SFME and ISFME methods, which resulted in chemical reactions such as oxidation, polymerization or degradation of some components in the essential oils, resulting in a darker colour. Furthermore, all extracted oils exhibited positive results in the phosphomolybdic acid reaction, indicating the presence of terpenes, esters, and alcohols within these oils.

### Analysis and comparison of different extraction processes for essential oil from Nanfeng mandarin peel

To investigate the impact of various extraction methods on the chemical composition of essential oils, we conducted a GC–MS data analysis. As presented in Table 6, we identified a total of 26, 27, and 28 volatile compounds in the oils extracted using the HD, SFME, and ISFME methods, respectively. The relative contents of oxygenated compounds like α-Terpinene, 4-(1-methylethenyl)−1-Cyclohexene-1-carboxaldehyde and Linalyl formate in the substances extracted by ISFME and SFME methods were significantly higher than that of HD method. This may be due to the reduced heating time required, which somewhat prevents the decomposition of oxygenated compounds (Jing et al. 2019).

**Table 6:** GC–MS analysis results of essential oil volatile compounds obtained by different extraction methods of Nanfeng mandarin orange peel

No	Retention time/min	Name	RI	CAS	Molecular formula	HD	SFME	ISFME
1	5.10	α-phellandrene	1004	99-83-2	C_10_H_16_	0.37	0.29	0.30
2	5.24	α-Pinene	937	80-56-8	C_10_H_16_	1.58	1.23	1.30
3	5.90	β-Phellandrene	1032	555-10-2	C_10_H_16_	0.31	0.31	0.30
4	5.98	β-pinene	981	127-91-3	C_10_H_16_	1.38	1.15	1.20
5	6.15	β-Myrcene	992	123-35-3	C_10_H_16_	2.61	2.51	2.54
6	6.35	Octyl aldehyde	1001	124-13-0	C_8_H_16_O	0.36	0.41	0.39
7	6.66	4-Carene	1023	29,050-33-7	C_10_H_16_	0.24	0.23	0.24
8	6.81	p-Cymene	1026	99-87-6	C_10_H_14_	0.18	0.13	0.14
9	6.93	D-Limonene	1033	138-86-3	C_10_H_16_	78.75	78.98	78.84
10	7.18	β-Ocimene	1051	13,877-91-3	C_10_H_16_	0.18	0.20	0.20
11	7.43	γ-Terpinene	1061	99-85-4	C_10_H_16_	9.34	9.56	9.58
12	7.57	1-Octanol	1075	111-87-5	C_8_H_18_O	*	0.05	0.04
13	7.97	α-Terpinene	1022	99-86-5	C_10_H_16_	0.49	0.49	0.49
14	8.12	Linalyl formate	1252	115-99-1	C_10_H_18_O	1.13	1.58	1.32
15	8.19	Nonanal	1083	124-19-6	C_9_H_18_O	0.09	*	0.09
16	9.10	Citronellal	1152	106-23-0	C_10_H_18_O	*	0.03	0.03
17	9.62	Terpinen-4-ol	1178	562-74-3	C_10_H_18_O	0.13	0.09	0.11
18	9.85	α-Terpineol	1193	98-55-5	C_10_H_18_0	0.31	0.33	0.32
19	10.03	Decanal	1205	112-31-2	C_10_H_20_O	0.41	0.43	0.45
20	10.46	2-Ethyl-6-Methylphenol	1236	1687-64-5	C_9_H_12_O	0.07	0.07	0.06
21	11.36	4-(1-methylethenyl)−1-Cyclohexene-1-carboxaldehyde	1264	2111-75-3	C_10_H_14_O	0.03	0.05	0.04
22	11.57	thymol	1296	89-83-8	C_10_H14O	0.06	0.15	0.12
23	11.82	Undecanal	1307	112-44-7	C_11_H_22_O	0.03	0.02	0.02
24	12.45	β-cadinene	1517	523-47-7	C_15_H_24_	0.08	0.07	0.07
25	13.36	β-elemene	1392	515-13-9	C_15_H_24_	0.05	0.04	0.04
26	13.52	Dodecanal	1409	112-54-9	C_12_H_24_O	0.07	0.06	0.07
27	14.98	α-Copaene	1374	3856-25-5	C_15_H_24_	0.16	0.15	0.15
28	15.31	α-Farnesene	1506	502-61-4	C_15_H_24_	1.57	1.37	1.55

The substance was not detected

Additionally, substances such as p-Cymene, ɑ-Pinene, β-pinene, and ɑ-phellandrene had much higher relative contents when extracted using the HD method compared to the ISFME and SFME methods. This can be attributed to thermal and hydrolytic effects. HD methods use large amounts of water and are time and energy consuming. Water is a polar solvent and accelerates many reactions, especially those with carbon cations as intermediates (Lucchesi et al. 2004). While analysing the extracts of ISFME and SFME methods, it was found that the proportion of olefins in the oil extracted by ISFME was 96.94%, of which D-limonene was 78.84%. Furthermore, the proportions of aldehydes, alcohols, phenols, and esters varied, accounting for 1.07%, 0.47%, 0.18%, and 1.32%, respectively. These findings reveal distinct differences in the retention and transformation of chemical components among the various extraction methods. This observation aligns with the results reported by Taktak et al. (2021), who identified 28 compounds in essential oil extracted from citrus peels using the SFME method, with limonene constituting 85.4% of the total. Essential oils extracted with ISFME contain much higher levels of oxygenates and much lower levels of monoterpene hydrocarbons than HD. The monoterpene hydrocarbons were less valuable than the oxygenates in terms of their contribution to the aroma of the essential oils. Conversely, oxygenates are highly aromatic and therefore have the highest value (Lucchesi et al. 2004).

**Fig. 7:**
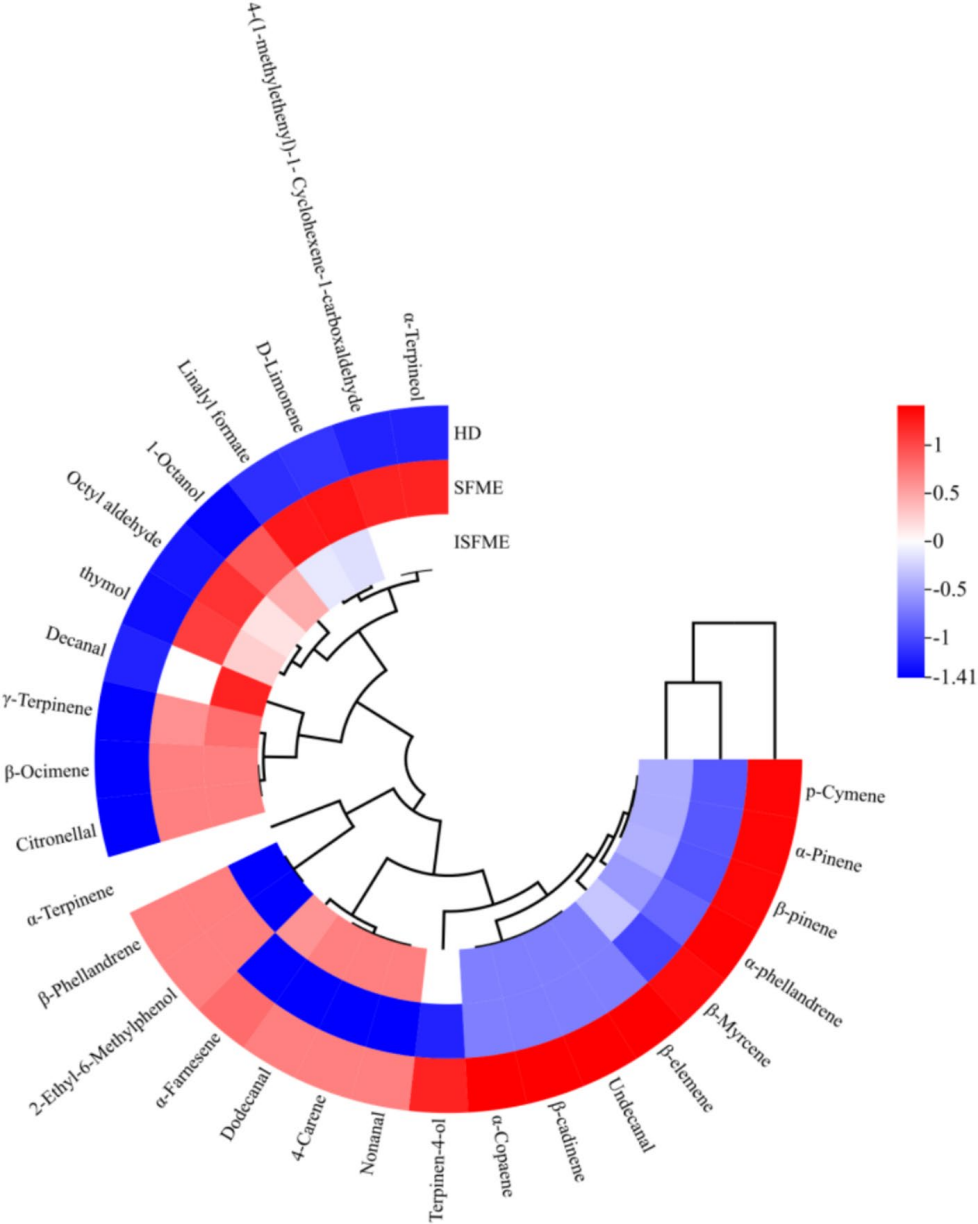
Cyclic clustering heatmap of the essential oil components of Nanfeng mandarin orange peel extracted by different methods

To enhance our understanding of the essential oil composition data and visualize the variations in compound proportions across different extraction methods, we present a circular heatmap accompanied by hierarchical cluster analysis in Fig. 7. The y-axis categorizes the essential oil components based on their relative contents, while the x-axis organizes the types and relative content differences of each component within the groups. Analysis of the x-axis clustering reveals that the SFME and ISFME methods form Cluster 1, while the HD method constitutes Cluster 2. This clustering suggests that the essential oil extraction outcomes from the SFME and ISFME methods are notably similar.

In light of the contemporary emphasis on environmental protection, this study also investigates the environmental impacts associated with various extraction methods. A comparison of CO_2_ emissions and electricity consumption, as shown in Table 7, reveals that the ISFME method is particularly commendable for its eco-friendliness. Specifically, the CO₂ emissions of ISFME (121.62 g) were much lower than those of HD (4800.26 g) and SFME (400.43 g), and were highly significantly different from both (*P* < 0.01), while the ISFME method consumed only 0.15 kW/h of electricity, which was significantly lower than those of HD and SFME. These findings not only highlight the ISFME method's significant advantages in efficiently extracting essential oils but also affirm its substantial potential for energy conservation, emission reduction, and environmental protection, aligning with the modern industry's demands for green and sustainable development.

**Table 7:** Comparison of and environmental impacts of Nanfeng honey orange essential oils

	HD	SFME	ISFME
Amount of CO_2_ released (g)	4800.26**	400.43**	121.62
Equipment power (kW/h)	6.03**	0.52**	0.15

“*”Indicates a significant effect on the results (*P* < 0.05)

“**”Indicates a highly significant effect on the results (*P* < 0.01)

### Antibacterial activity of essential oils

**Fig. 8:**
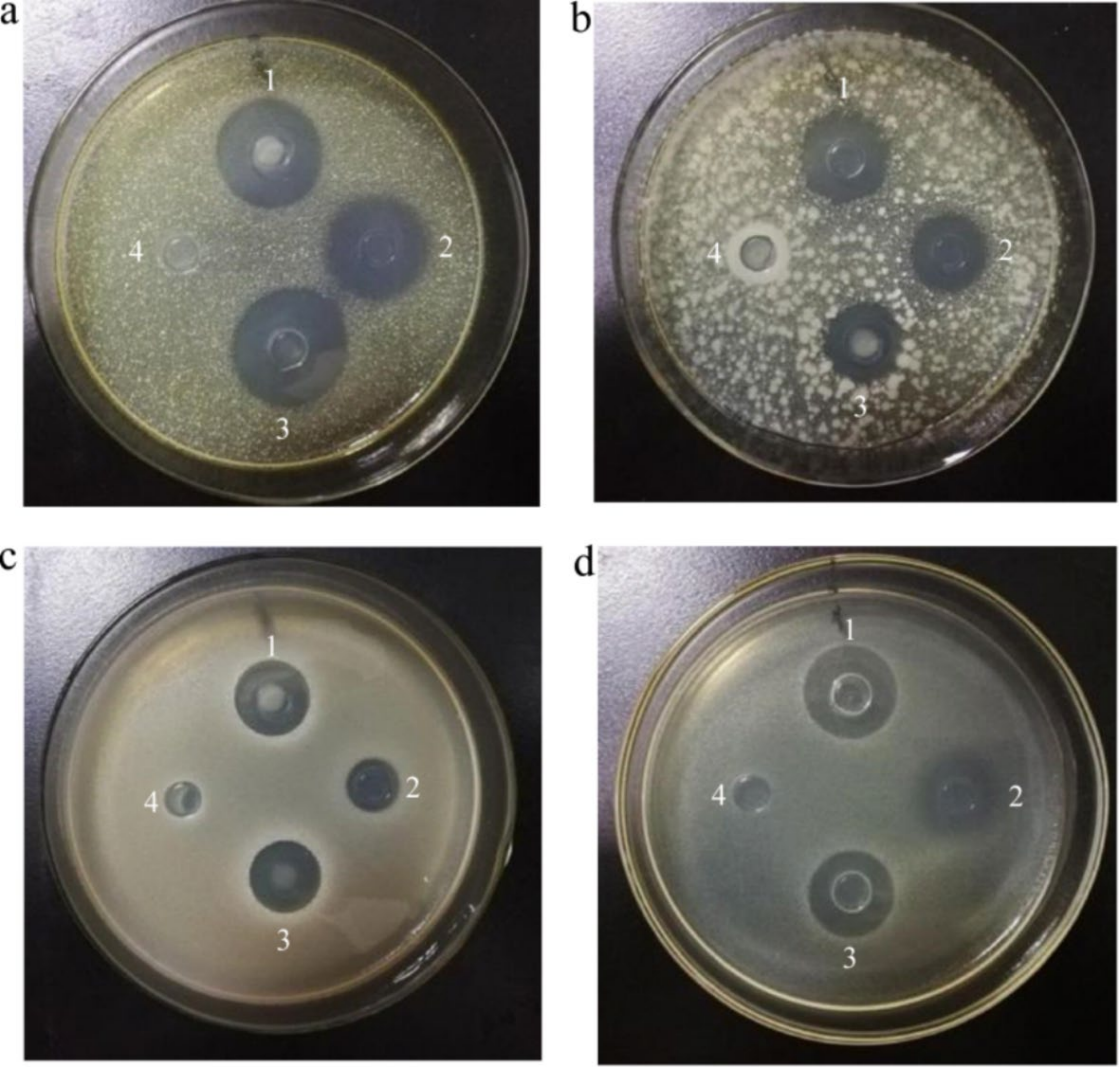
Bacteriostatic effect of essential oils on six pathogenic bacteria. undiluted essential oil (1,3), positive control: Chloramphenicol (2), negative control: pure water (4). **a**: *Staphylococcus aureus*, **b**: *Bacillus cereus*, **c**: *Salmonella typhimurium*, **d**: *Shigella flexneri*

The inhibitory effects of essential oils derived from Nanfeng mandarin, extracted using the ISFME method, on four pathogenic bacteria (including Gram-positive bacteria *Staphylococcus aureus* and *Bacillus cereus*; Gram-negative bacteria *Salmonella typhimurium* and *Shigella flexneri*) are illustrated in Fig. 8 and detailed in Table 8. The results indicated that the essential oils exhibited significant inhibitory effects against all four pathogenic bacteria. To further explore the antibacterial activity of these essential oils, various gradients were established to determine the MIC values, and the results are shown in Table 8. The MIC values demonstrated that the order of inhibitory effects of the essential oils on the pathogenic bacteria was as follows: *Staphylococcus aureus* > *Salmonella typhimurium* > *Shigella flexneri* > *Bacillus cereus*. These findings suggest that the essential oils of Nanfeng mandarin possess broad-spectrum inhibitory capabilities against pathogenic bacteria, consistent with the results reported by Denkova-Kostova et al. (2021) regarding their bacteriostatic effects.

**Table 8:** Diameter and MIC values of the inhibitory effect of essential oils on pathogenic bacteria

Pathogenic bacteria	Inhibition (mm, mean ± SD)	MIC (mg/mL)
*Staphylococcus aureus*	23.3±1.7	3.2
*Bacillus cereus*	17.3±1.7	6.3
*Salmonella typhimurium*	16.3±0.7	4.4
*Shigella flexneri*	17.6±1.4	3.5

The diameter data in the table are the mean (±SD) of the size of the inhibition circle, 0: no inhibition circle. The diameter of the Oxford cup is 7.8 mm

## Conclusions

This study investigates the application of SFME and ISFME for extracting essential oils from the peel of Nanfeng mandarin, comparing the oil composition with those obtained through traditional extraction methods, namely HD. Optimal parameters were established through single-factor experiments and response surface optimization. For the SFME method, the optimal conditions were identified as a power of 660 W, an extraction time of 20 min, and a moisture content of 60%. In contrast, the optimal conditions for ISFME were determined to be a moisture content of 50%, a microwave power of 380 W, a single reaction time of 6 min, an interval of 15 min between reactions, and a total of 4 reaction cycles. Both SFME methods, under conditions that ensure low reaction times, yielded significantly higher essential oil outputs compared to the HD, while also consuming less energy. This highlights the superior efficiency of SFME over traditional extraction techniques. Analysis of GC–MS data and clustering heatmaps demonstrated that the types of essential oil compounds extracted by the three methods were identical; however, the relative contents of essential oil extracts from ISFME and SFME were more closely aligned. Despite similar reaction times, extraction rates, and oil qualities between ISFME and SFME, the power requirement for ISFME was only 380 W, significantly lower than the 660 W required for SFME. Moreover, ISFME's CO_2_ emissions and electricity consumption were merely one-third of those of SFME. This indicates that ISFME not only provides high efficiency and quality but also realizes significant energy savings and reductions in emission.

The ISFME method has demonstrated excellent advantages in large-scale industrial production. The method not only maintains the traditional advantages of high extraction rate and high quality essential oil output, but also achieves a major breakthrough in energy saving, emission reduction and environmental protection. Compared with the traditional hydrodistillation (HD) and SFME, the ISFME method significantly reduces the energy consumption based on the optimisation of the reaction cycle and microwave power, with the power requirement of 380 W much lower than that of SFME's 660 W, and the design of the reaction spacing, which further reduces the unnecessary energy consumption. At the same time, the CO_2_ emissions and power consumption of the ISFME method are only one-third of those of SFME, which perfectly fits the current global demand for sustainable development and green manufacturing. The wide application of this green technology will not only promote the development of the essential oil extraction industry in a more efficient and environmentally friendly direction, but also bring revolutionary changes to the entire industrial chain, leading the industry towards a green and sustainable future. Therefore, ISFME method is undoubtedly the preferred solution in the field of industrial extraction of essential oils and has a broad development prospect.

## Supplementary Information

Supplementary material 1.

## Data Availability

Not applicable.

## References

[CR1] Arena ME, Alberto MR, Cartagena E (2021) Potential use of *Citrus* essential oils against acute respiratory syndrome caused by coronavirus. J Essent Oil Res 33:330–341. 10.1080/10412905.2021.1912839

[CR2] Bai J, Li J, Chen Z, Bai X, Yang Z, Wang Z, Yang Y (2023) Antibacterial activity and mechanism of clove essential oil against foodborne pathogens. LWT 173:114249. 10.1016/j.lwt.2022.114249

[CR3] Benmoussa H, Elfalleh W, He S, Romdhane M, Benhamou A, Chawech R (2018) Microwave hydrodiffusion and gravity for rapid extraction of essential oil from Tunisian cumin (*Cuminum cyminum* L.) seeds: optimization by response surface methodology. Ind Crops Prod 124:633–642. 10.1016/j.indcrop.2018.08.036

[CR4] Bhandari DP, Poudel DK, Satyal P, Khadayat K, Dhami S, Aryal D, Chaudhary P, Ghimire A, Parajuli N (2021) Volatile compounds and antioxidant and antimicrobial activities of selected citrus essential oils originated from Nepal. Molecules 26:6683. 10.3390/molecules26216683 34771092 10.3390/molecules26216683 PMC8588556

[CR5] Bustamante J, Van Stempvoort S, García-Gallarreta M, Houghton JA, Briers HK, Budarin VL, Matharu AS, Clark JH (2016) Microwave assisted hydro-distillation of essential oils from wet citrus peel waste. J Clean Prod 137:598–605. 10.1016/j.jclepro.2016.07.108

[CR6] Cebi N, Erarslan A (2023) Determination of the antifungal, antibacterial activity and volatile compound composition of citrus bergamia peel essential oil. Foods 12:203. 10.3390/foods12010203 36613419 10.3390/foods12010203 PMC9818623

[CR7] Chen Y, Gu X, Huang S, Li J, Wang X, Tang J (2010) Optimization of ultrasonic/microwave assisted extraction (UMAE) of polysaccharides from Inonotus obliquus and evaluation of its antitumor activities. Int J Biol Macromol 46:429–435. 10.1016/j.ijbiomac.2010.02.003 20149817 10.1016/j.ijbiomac.2010.02.003

[CR8] Chen H, Gu Z, Yang L, Yang R, Ji Y, Zeng Q, Xiao F, Huang P (2021) Optimization extraction of rosemary essential oils using hydrodistillation with extraction kinetics analysis. Food Sci Nutr 9:6069–6077. 10.1002/fsn3.2549 34760238 10.1002/fsn3.2549 PMC8565215

[CR9] Chumnanpaisont N, Niamnuy C, Devahastin S (2014) Mathematical model for continuous and intermittent microwave-assisted extraction of bioactive compound from plant material: extraction of β-carotene from carrot peels. Chem Eng Sci 116:442–451. 10.1016/j.ces.2014.05.010

[CR10] Denkova-Kostova R, Teneva D, Tomova T, Goranov B, Denkova Z, Shopska V, Slavchev A, Hristova-Ivanova Y (2021) Chemical composition, antioxidant and antimicrobial activity of essential oils from tangerine ( *Citrus reticulata* L.), grapefruit ( *Citrus paradisi* L.), lemon ( *Citrus lemon* L.) and cinnamon ( *Cinnamomum zeylanicum* Blume). Zeitschrift Für Naturforschung C 76:175–185. 10.1515/znc-2020-0126 10.1515/znc-2020-0126 33909955

[CR11] Desai MA, Parikh J (2015) Extraction of essential oil from leaves of lemongrass using microwave radiation: optimization, comparative, kinetic, and biological studies. ACS Sustain Chem Eng 3:421–431. 10.1021/sc500562a

[CR12] Falleh H, Ben Jemaa M, Saada M, Ksouri R (2020) Essential oils: a promising eco-friendly food preservative. Food Chem 330:127268. 10.1016/j.foodchem.2020.127268 32540519 10.1016/j.foodchem.2020.127268

[CR13] Handayani AD, Sutrisno I, Ismadji N (2008) Extraction of astaxanthin from giant tiger (*Panaeus monodon*) shrimp waste using palm oil: studies of extraction kinetics and thermodynamic. Biores Technol 99:4414–4419. 10.1016/j.biortech.2007.08.028 10.1016/j.biortech.2007.08.028 17911016

[CR14] Hosseini SS, Khodaiyan F, Yarmand MS (2016) Optimization of microwave assisted extraction of pectin from sour orange peel and its physicochemical properties. Carbohyd Polym 140:59–65. 10.1016/j.carbpol.2015.12.051 10.1016/j.carbpol.2015.12.051 26876828

[CR15] Jing CL, Huang RH, Su Y, Li YQ, Zhang CS (2019) Variation in chemical composition and biological activities of flos chrysanthemi indici essential oil under different extraction methods. Biomolecules 9:518. 10.3390/biom9100518 31546663 10.3390/biom9100518 PMC6843213

[CR16] Joseph SM, Dev ARA (2023) Unveiling the volatile chemical variations of Annona essential oils and its associated pharmacological activities. J Mol Struct 1292:136082. 10.1016/j.molstruc.2023.136082

[CR17] Junxing Li, Aiqing M, Gangjun Zhao, Liu Xiaoxi Wu, Haibin LJ, Hao G, Xiaoming Z, Liting D, Chengying Ma (2022) Assessment of the ‘taro-like’ aroma of pumpkin fruit (*Cucurbita**moschata* D) via E-nose, GC–MS and GC-O analysis. Food Chem 15:100435. 10.1016/j.fochx.2022.100435 10.1016/j.fochx.2022.100435 PMC9532776 36211734

[CR18] Kratchanova M, Pavlova E, Panchev I (2004) The effect of microwave heating of fresh orange peels on the fruit tissue and quality of extracted pectin. Carbohyd Polym 56:181–185. 10.1016/j.carbpol.2004.01.009

[CR19] Kumar C, Joardder MUH, Farrell TW, Karim MA (2016) Multiphase porous media model for intermittent microwave convective drying (IMCD) of food. Int J Therm Sci 104:304–314. 10.1016/j.ijthermalsci.2016.01.018

[CR20] Kusuma HS, Altway A, Mahfud M (2018) Solvent-free microwave extraction of essential oil from dried patchouli (*Pogostemon cablin* Benth) leaves. J Ind Eng Chem 58:343–348. 10.1016/j.jiec.2017.09.047

[CR21] Li Y, Liu S, Zhao C, Zhang Z, Nie D, Tang W, Li Y (2022) The chemical composition and antibacterial and antioxidant activities of five citrus essential oils. Molecules 27:7044. 10.3390/molecules27207044 36296637 10.3390/molecules27207044 PMC9607008

[CR22] Liu Z, Deng B, Li S, Zou Z (2018) Optimization of solvent-free microwave assisted extraction of essential oil from *Cinnamomum**camphora* leaves. Ind Crops Prod 124:353–362. 10.1016/j.indcrop.2018.08.016

[CR23] Lu X, Zhao C, Shi H, Liao Y, Xu F, Du H, Xiao H, Zheng J (2023) Nutrients and bioactives in citrus fruits: different citrus varieties, fruit parts, and growth stages. Crit Rev Food Sci Nutr 63:2018–2041. 10.1080/10408398.2021.1969891 34609268 10.1080/10408398.2021.1969891

[CR24] Lucchesi ME, Chemat F, Smadja J (2004) Solvent-free microwave extraction of essential oil from aromatic herbs: comparison with conventional hydro-distillation. J Chromatogr A 1043:323–327. 10.1016/j.chroma.2004.05.083 15330107 10.1016/j.chroma.2004.05.083

[CR25] Ma C, Yang L, Zu Y, Liu T (2012) Optimization of conditions of solvent-free microwave extraction and study on antioxidant capacity of essential oil from *Schisandra**chinensis* (Turcz.) Baill. Food Chem 134:2532–2539. 10.1016/j.foodchem.2012.04.080 23442721 10.1016/j.foodchem.2012.04.080

[CR26] Mahato N, Sharma K, Koteswararao R, Sinha M, Baral E, Cho MH (2019) Citrus essential oils: extraction, authentication and application in food preservation. Crit Rev Food Sci Nutr 59:611–625. 10.1080/10408398.2017.1384716 28956626 10.1080/10408398.2017.1384716

[CR27] Mollaei S, Sedighi F, Habibi B, Hazrati S, Asgharian P (2019) Extraction of essential oils of *Ferulago**angulata* with microwave-assisted hydrodistillation. Ind Crops Prod 137:43–51. 10.1016/j.indcrop.2019.05.015

[CR28] Pei TH, Zhao YJ, Wang SY, Li XF, Sun CQ, Shi SS, Xu ML, Gao Y (2023) Preliminary study on insecticidal potential and chemical composition of five rutaceae essential oils against thrips flavus (*Thysanoptera*: *Thripidae*). Molecules 28:2998. 10.3390/molecules28072998 37049761 10.3390/molecules28072998 PMC10095842

[CR29] Peng X, Yang X, Gu H, Yang L, Gao H (2021) Essential oil extraction from fresh needles of *Pinus**pumila* (Pall) Regel using a solvent-free microwave-assisted methodology and an evaluation of acetylcholinesterase inhibition activity in vitro compared to that of its main components. Industrial Crops Prod 167:113549. 10.1016/j.indcrop.2021.113549

[CR30] Prommaban A, Chaiyana W (2022) Microemulsion of essential oils from citrus peels and leaves with anti-aging, whitening, and irritation reducing capacity. J Drug Delivery Sci Technol 69:103188. 10.1016/j.jddst.2022.103188

[CR31] Pu D, Wei L, Wei L, Li H, Zhu M, Lu Q, Bao Y, Zu Y (2023) Extraction and *in vitro* active evaluation of essential oil of *Acorus tatarinowii* Schott rhizome rich in β-asarone using enzymatic pretreatment and solvent-free microwave-assisted method. J Essential Oil Bearing Plants 26:1563–1575. 10.1080/0972060X.2023.2277901

[CR32] Silveira PG, Corrêa JLG, Silva CRDP, Macedo LL, Gonçalves WS, Júnior IP (2024) Innovative strategies in yacon drying: Ethanol pretreatment and intermittent microwave drying. J Food Sci 89:4941–4952. 10.1111/1750-3841.17254 39013009 10.1111/1750-3841.17254

[CR33] Singh Chouhan KB, Tandey R, Sen KK, Mehta R, Mandal V (2019) A unique model of gravity assisted solvent free microwave based extraction of essential oil from mentha leaves ensuring biorefinery of leftover waste biomass for extraction of nutraceuticals: towards cleaner and greener technology. J Clean Prod 225:587–598. 10.1016/j.jclepro.2019.03.325

[CR34] Taktak O, Ben Youssef S, Abert Vian M, Chemat F, Allouche N (2021) Physical and chemical influences of different extraction techniques for essential oil recovery from *Citrus sinensis* peels. J Essential Oil Bearing Plants 24:290–303. 10.1080/0972060X.2021.1925596

[CR35] Talib Al-Sudani B, Khoshkalampour A, Kamil MM, Al-Musawi MH, Mohammadzadeh V, Ahmadi S, Ghorbani M (2024) A novel antioxidant and antimicrobial food packaging based on Eudragit ®/collagen electrospun nanofiber incorporated with bitter orange peel essential oil. LWT 193:115730. 10.1016/j.lwt.2024.115730

[CR36] Teigiserova DA, Tiruta-Barna L, Ahmadi A, Hamelin L, Thomsen M (2021) A step closer to circular bioeconomy for citrus peel waste: a review of yields and technologies for sustainable management of essential oils. J Environ Manage 280:111832. 10.1016/j.jenvman.2020.111832 33360259 10.1016/j.jenvman.2020.111832

[CR37] Tsai ML, Lin CD, Khoo K, Wang MY, Kuan TK, Lin WC, Zhang YN, Wang YY (2017) Composition and bioactivity of essential oil from citrus grandis (L) *Osbeck* ‘Mato Peiyu’ Leaf. Molecules 22:2154. 10.3390/molecules22122154 29206180 10.3390/molecules22122154 PMC6149744

[CR38] Vasta JV, Sherma J (2008) Comparison of spraying, dipping, and the derivapress for postchromatic derivatization with phosphomolybdic acid in the detection and quantification of neutral lipids by high-performance thin-layer chromatography. Acta Chromatogr 20:15–23. 10.1556/AChrom.20.2008.1.2

[CR39] Vorhauer N, Tretau A, Bück A, Prat M (2019) Microwave drying of wet clay with intermittent heating. Drying Technol 37:664–678. 10.1080/07373937.2018.1547740

[CR40] Wang S, Ding S, Meng K, Liu X, Wang Y, Wang X, Qin X, Luo H, Yao B, Huang H, Tu T (2022) Preparation of methyl-esterified pectin oligosaccharides with antibacterial activity using fungus-derived bifunctional pectinase. J Clean Prod 333:130110. 10.1016/j.jclepro.2021.130110

[CR41] Wei L, Yu X, Li H, Zhu M, Pu D, Lu Q, Bao Y, Zu Y (2023) Optimization of solvent-free microwave extraction of essential oil from the fresh peel of Citrus medica L var arcodactylis Swingle by response surface methodology, chemical composition and activity characterization. Scientia Horticulturae 309:111663. 10.1016/j.scienta.2022.111663

[CR42] Zhou L, Hao M, Min T, Bian X, Du H, Sun X, Zhu Z, Wen Y (2023) Kaolin incorporated with thyme essential oil for humidity-controlled antimicrobial food packaging. Food Packag Shelf Life 38:101106. 10.1016/j.fpsl.2023.101106

