# Supplementary figures and images for: Sustainable and green extraction of citrus peel essential oil using intermittent solvent-free microwave technology
Source: Bioresour Bioprocess. 2025 May 29;12(1):48. doi: 10.1186/s40643-025-00885-6 (PMC12122996; doi:10.1186/s40643-025-00885-6)

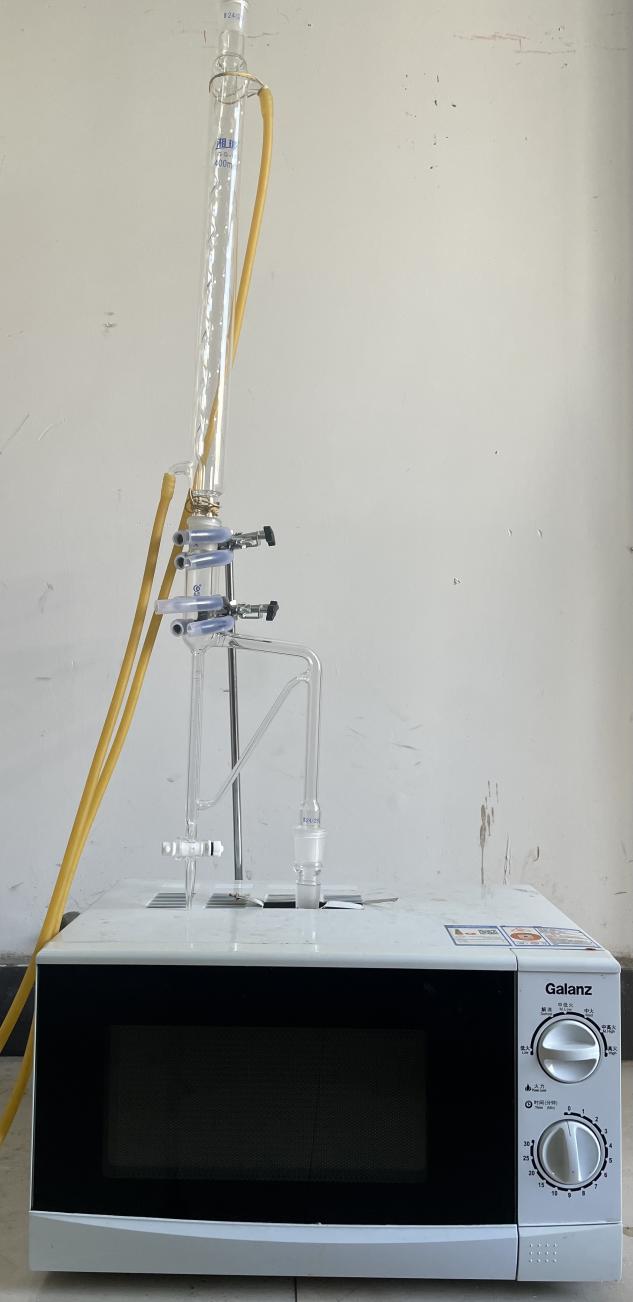


**Fig. S1.** Microwave extraction device


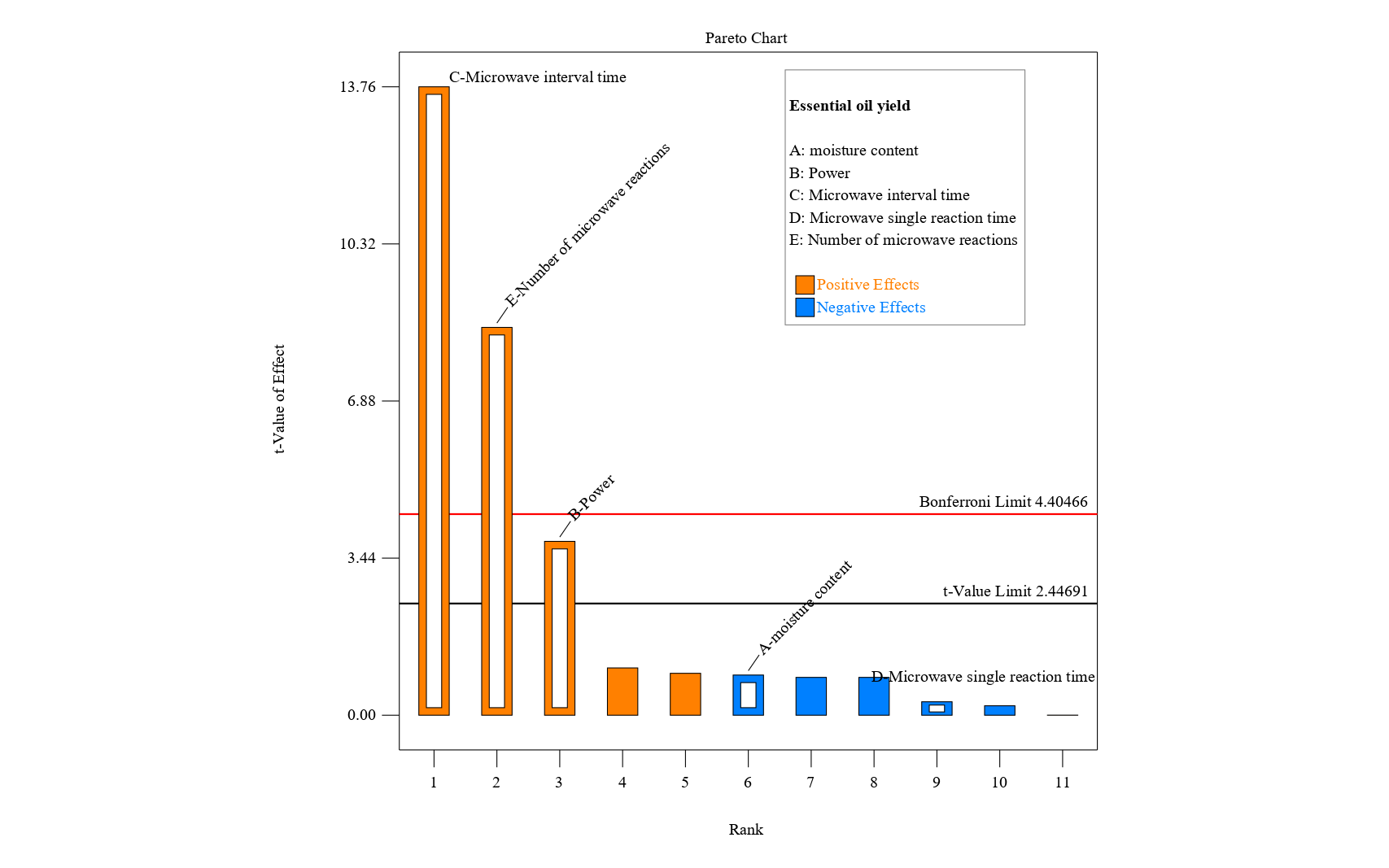


**Fig. S2.** Pareto Chart of the Standardized Effects (α = 0.05)

Supplement: Supplementary file 1 — Supplementary material 1. [file 40643_2025_885_MOESM1_ESM.docx]
